# Hormone Receptor Positive/HER2 Negative Breast Carcinoma: Association of *PIK3CA* Mutational Status with PD-L1 and Tumor Cell Microenvironment and Their Prognostic Significance

**DOI:** 10.3390/ijms26199489

**Published:** 2025-09-28

**Authors:** Danijel Lopac, Emina Babarović, Justin Hagen, Petra Valković Zujić, Damir Grebić, Ita Hadžisejdić

**Affiliations:** 1 Clinic for Orthopedic Surgery, 51415 Lovran, Croatia; dlopac@gmail.com; 2 Faculty of Medicine, University of Rijeka, 51000 Rijeka, Croatia; emina.babarovic@uniri.hr (E.B.); jhagen@student.uniri.hr (J.H.); petra.valkovic.zujic@uniri.hr (P.V.Z.); damir.grebic@uniri.hr (D.G.); 3 Clinical Department of Pathology and Cytology, Clinical Hospital Center Rijeka, 51000 Rijeka, Croatia; 4 Clinical Department of Diagnostic and Intervetional Radiology, Clinical Hospital Center Rijeka, 51000 Rijeka, Croatia; 5 Department of Surgery, Clinical Hospital Center Rijeka, 51000 Rijeka, Croatia

**Keywords:** breast cancer, *PIK3CA* mutation, PD-L1, immune infiltrate

## Abstract

Novel research data in different cancer types indicate that mutations within *PIK3CA* might serve as a biomarker of an improved response to immune therapy. Therefore, the aim of this study was to evaluate and examine possible differences in the tumor microenvironment composition and PD-L1 expression as well the prognostic significance of CD4, CD8, CD68, and CD163 in *PIK3CA* mutated and non-mutated hormone receptor positive and HER2 negative (HR+/HER2−) breast carcinoma. Breast carcinoma tissue was analyzed by Cobas *PIK3CA* mutation test for the presence of *PIK3CA* mutation and immunohistochemistry was applied to assess PD-L1 expression and CD4, CD8, CD68, and CD163 infiltration within tumor. Statistically significant association was observed between PD-L1 expression and the presence of *PIK3CA* exon 20 mutation (*p* = 0.044), with PD-L1–positive patients predominantly harboring this mutation. Tumors harboring *PIK3CA* mutations exhibited moderate to strong statistically significant positive correlations between PD-L1 expression and infiltration by CD8 cells (rs = 0.462, *p* = 0.0027), CD68 cells (rs = 0.398, *p* = 0.0134), and CD163 cells (rs = 0.617, *p* < 0.0001). In patients with *PIK3CA* mutation and exon 20 *PIK3CA* mutation there was statistically significant longer survival without recurrence (*p* = 0.026 and *p* = 0.041, respectively). Research regarding PD-L1 expression, immune cells and *PIK3CA* mutations might have an impact on how to determine therapeutic approaches for patients with HR+/HER2− breast carcinoma.

## 1. Introduction

Breast cancer is the leading cause of cancer-related death among women despite early diagnosis and many advancements in treatment. Additionally, many breast cancer patients are unable to achieve durable and effective treatment response. Immune therapy is emerging as one of the promising options for management of many types of cancer and one of them is breast carcinoma, although it was considered to be a malignancy of low immunogenicity. Immune infiltration within the tumor microenvironment (TME) significantly influences the outcome of immunotherapy and other anti-tumor treatments [1,2]. Based on the composition and functional state of the immune cells within the TME, they can either boost or restrain tumor growth. High levels of tumor-infiltrating lymphocytes (TILs) are often associated with better responses to immune checkpoint inhibitors and improved patient outcomes [1,3]. Concerning the predictive and prognostic role of TILs in early hormone receptor positive/human epidermal growth factor-2 negative (HR+/HER2−) breast carcinoma, conflicting results have been highlighted: a significant association between high TILs and a worse prognosis has emerged in some studies, while other authors failed to demonstrate the prognostic significance of TILs [4,5].

In breast cancer, programmed cell death ligand 1 (PD-L1) expression levels are typically lower than in other solid tumors, with an expression rate of approximately 10–20% [6,7]. PD-L1 expression in early breast carcinoma is variable, based on tumor stage and molecular subtypes, where triple negative breast carcinomas (TNBC) show the highest percentage of PD-L1 positivity (around 45–55%), followed by HER2+ tumors (around 30%) while, PD-L1 is rarely expressed in HR+/HER2− breast carcinomas (around 0–10% of cases) [5]. Due to different immunohistochemistry (IHC) clones, cut-off points and scoring systems, the prognostic role of PD-L1 expression in breast carcinoma is still controversial. Based on TIL count and PD-L1 expression, primary breast cancer tumors show higher immunogenicity than the metastatic tumor samples [8]. *PIK3CA* is the most frequently mutated gene in breast cancer but its relevance to the breast cancer prognosis remains controversial [9]. Increasing evidence suggests that in addition to the direct proliferative effects on tumor cells, the PI3K-AKT-mTOR pathway is involved in creating an immunosuppressive TME by enhanced expression of PD-L1, recruitment and differentiation of myeloid-derived suppressor cells (MDSCs) and Tregs into the tumor, and secretion of suppressive cytokines to impair stimulation of macrophages and dendritic cells and the migration, expansion, functionality, and memory development of T cells [10].

*PIK3CA*-mutations are considered an early event in breast cancer development since they were detected even in small tumors as well as in non-invasive precursor lesions, like ductal carcinoma in situ (DCIS) [11]. Reported mutation rates for *PIK3CA* in breast carcinoma range from 18% to 40% [12]. The highest incidence of mutations, in the *PIK3CA* gene, is found in the most common subtype of breast cancer, in the HR+/HER2− breast carcinoma [13]. Approximately 80% of *PIK3CA* mutations occur in the helical and kinase domains, with E542K and E545K in exon 9 as well as H1047R and H1047L in exon 20, as the most common variants [14]. *PIK3CA* exon 20 mutations are associated with higher p-ERK1/2 levels that belong to the mitogen activated protein (MAP) kinases pathway, and tumors with a *PIK3CA* exon 9 mutation are associated with higher p-AKT and p-ERK1/2, but not with p-p70S6K [15]. Studies indicate poorer survival and development of resistance to hormonal therapy in patients with metastatic breast cancer who are HR+/HER2− and have a *PIK3CA* mutations [16]. Some studies indicate that exon 9 mutations are associated with a slightly worse prognosis [17]. While *PIK3CA*-mutated HR+/HER2− metastatic breast carcinoma is generally associated with poor outcome and resistance to chemotherapy Mosele F et al. showed that patients with *PIK3CA* mutated TNBC had improved overall survival [18]. They proposed that this finding might be explained by enrichment of *PIK3CA* mutations in the luminal breast carcinomas that subsequently lost hormone expression in the metastatic setting [18]. Also, mutations of the *PIK3CA* gene can influence changes in the TME and alter the body’s immune response. For example, in colon and stomach cancer, studies have shown the connection of *PIK3CA* gene mutation with increased infiltration by T cytotoxic lymphocytes, higher expression of PD-L1 on tumor cells and greater effectiveness of immunotherapy [19]. A study by Sobral-Leite et al. indicated that luminal breast cancer with high CD8 infiltration was associated with unfavorable outcome and that PI3K pathway alterations might be associated with the composition of the tumor microenvironment [20]. Also, a study by Jiang W et al. showed that *PIK3CA* mutation in cervical carcinoma creates an immune-suppression environment by increasing PD-L1 transcriptional expression and repressing the differentiation of CD8 T cells [21]. They also showed that PI3Kα inhibitor significantly enhances the anti-tumor efficacy of PD-1 blockade in cell derived xenografts and patient derived xenografts indicating that *PIK3CA* mutations may be predictors of cervical cancer response to PD-1 blockade [21].

Several characteristics of *PIK3CA* mutations in breast cancer have been observed, including a strong association with expression of the estrogen receptor (ER), a lack of an association with robust activation of the classical PI3K pathway, as well as a relatively good prognosis for patients with mutations compared with their wild-type counterparts [22,23].

Because there is increasing evidence that *PIK3CA* mutation alone or in combination with PD-L1 positivity may better predict the efficacy of immune checkpoint blockades, there is need to better characterize relationship between *PIK3CA* mutation, PD-L1 expression and TME in the HR+/HER2−, the most frequent type of breast carcinoma. Therefore, the aim of this study is to evaluate and examine possible differences in the TME composition and PD-L1 expression as well the prognostic significance of CD4, CD8, CD68, and CD163 in *PIK3CA* mutated and non-mutated HR+/HER2− breast carcinoma.

## 2. Results

### 2.1. Study Cohort

In this study group, out of 123 samples, 43.1% (53/123) had *PIK3CA* mutation with the majority having mutations in exon 20 (26.8%) and exon 9 (16.3%). Mutations in other exons covered by the used real time PCR assay were not found. Also, when looking at the PD-L1 expression in our study group, we found 9.8% (12/123) positive cases (Table 1). In the group with the *PIK3CA* mutation, there was 19% (8/42) of PD-L1 positive tumors, while tumors without a *PIK3CA* mutation were PD-L1 positive in only 6.7% (3/45) of cases (Table 3). Also, more tumors with exon 20 *PIK3CA* mutation were PD-L1 positive 25.9% (7/27) in comparison to 6.7% (1/15) PD-L1 positive tumors, which had exon 9 *PIK3CA* mutation (Table 3). The more detailed tumor characteristics of the study group are shown in Table 1.

### 2.2. Association of Clinical and Pathological Characteristics with PIK3CA Mutational Status

When looking into tumor characteristics in comparison to *PIK3CA* mutational status, we only found association with the multifocally and bilateral presence of breast carcinoma, and that was at the level of statistical trend (*p* = 0.089 and *p* = 0.059, respectively) (Table 2). However, when looking at the tumor characteristics depending on the exon 9 and exon 20 *PIK3CA* mutation, there was statistically significant association with Ki-67 proliferating index with much more cases with exon 20 mutation with a Ki-67 lower than 20% (*p* = 0.041). Also, there was an association of exon 9 and exon 20 *PIK3CA* mutation distribution with more frequently found tumors having low and intermediate histological grades at the level of statistical trend (*p* = 0–055). In addition, more tumors with exon 20 *PIK3CA* mutation were without metastasis/recurrence, but this finding was also at the level of the statistical trend (*p* = 0.097) (Table 2).

### 2.3. CD4, CD8, CD68, CD163 Distribution and PD-L1 Expression in Non-Mutated and Mutated PIK3CA Carcinomas

Comparison of the TME composition and the infiltration of CD4, CD8, CD68, and CD163 between tumor tissues with and without *PIK3CA* mutation revealed no statistically significant differences in their distribution. The comparison was performed using both median values and receiver operating characteristic (ROC) curve-derived cut-off points for the evaluated immune cell populations, yielded no statistically significant results (Table 3). Further on, a comparison of the PD-L1 expression between *PIK3CA* mutated and non-mutated tumors did not show a statistically significant difference (*p* = 0.110). A statistically significant association was observed between PD-L1 expression and the presence of a *PIK3CA* exon 20 mutation (*p* = 0.044), with PD-L1–positive patients predominantly harboring this mutation (Table 3). When comparing the composition of immune cells in the whole group, depending on metastasis/recurrence, there was statistically significant distribution of CD163 macrophages where the majority of patients with low CD163 infiltrate had metastasis/recurrence (*p* = 0.002) (Table 4). Also, a statistical trend was noticed in CD4 and PD-L1 distribution between these groups where tumors with high CD4 infiltrate and positive PD-L1 did not have metastasis/recurrence (*p* = 0.096 and *p* = 0.065, respectively) (Table 4). However, depending on *PIK3CA* mutational status, tumors without *PIK3CA* mutation and high CD4 infiltrate were less likely to have metastasis (*p* = 0.039) and low CD163 tumors with *PIK3CA* mutation more frequently had metastasis (*p* = 0.036) (Table 4). All other comparisons were not statistically significant. In addition, the statistical analysis and comparison of immune cell composition in luminal A and luminal B breast carcinoma with and without *PIK3CA* mutation was performed but there was no significant result (Supplementary Data Table S1).

### 2.4. Correlation of PD-L1 Expression Depending on PIK3CA Mutational Status

In the overall study cohort, PD-L1 expression demonstrated a weak statistically significant positive correlation with infiltration by CD4 cells (rs = 0.203, *p* = 0.051), CD8 cells (rs = 0.312, *p* = 0.0026), and CD68 cells (rs = 0.238, *p* = 0.023). In tumors lacking *PIK3CA* mutations, PD-L1 expression correlated negatively with CD163 cell infiltration (rs = –0.317, *p* = 0.052), while all other correlations were not statistically significant. In contrast, tumors harboring *PIK3CA* mutations exhibited moderate to strong statistically significant positive correlations between PD-L1 expression and infiltration by CD8 cells (rs = 0.462, *p* = 0.0027), CD68 cells (rs = 0.398, *p* = 0.0134), and CD163 cells (rs = 0.617, *p* < 0.0001) (Table 5).

### 2.5. Survival Analysis

**Figure 1:**
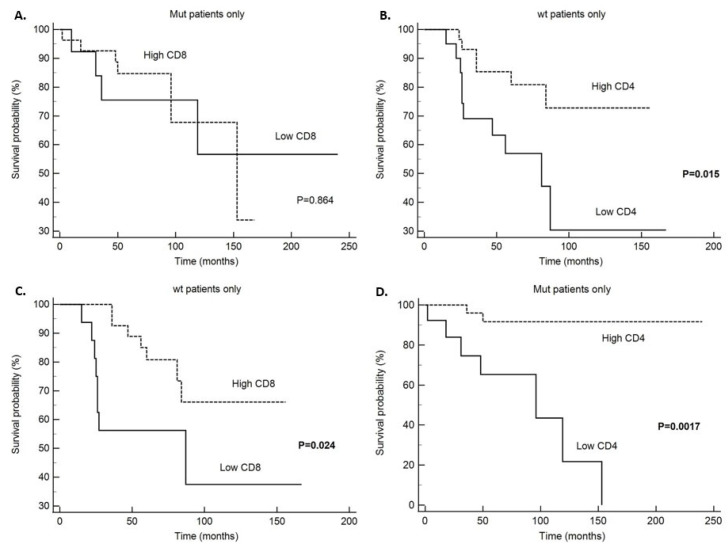
Disease specific survival (DSS); (**A**)—CD4 infiltrate in tumors without *PIK3CA* mutation (wt tumors), (**B**)—CD4 infiltrate in *PIK3CA* mutated tumors, (**C**)—CD8 infiltrate in tumors without *PIK3CA* mutation (wt tumors), (**D**)—CD8 infiltrate in *PIK3CA* mutated tumors.

**Figure 2:**
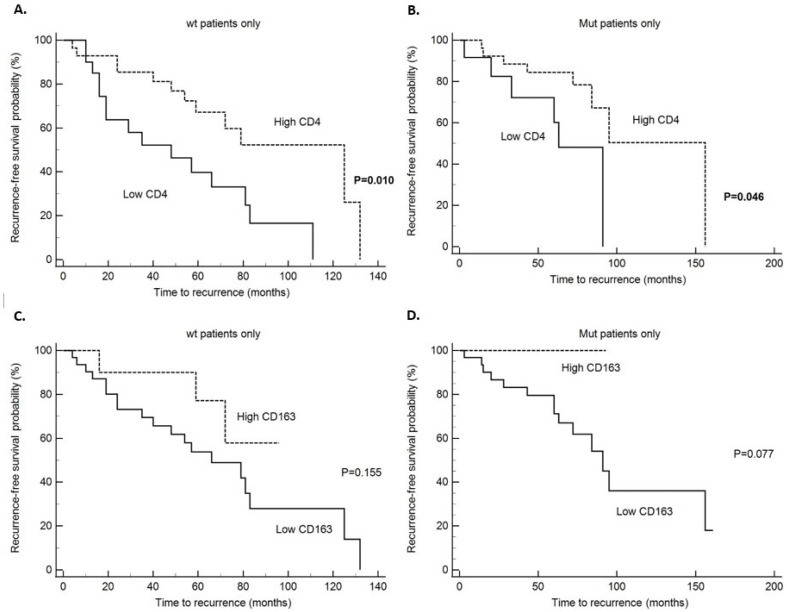
Disease-free survival (DFS); (**A**)—CD4 infiltrate in tumors without *PIK3CA* mutation (wt tumors), (**B**)—CD4 infiltrate in *PIK3CA* mutated tumors, (**C**)—CD163 infiltrate in tumors without *PIK3CA* mutation (wt tumors), (**D**)—CD163 infiltrate in *PIK3CA* mutated tumors.

**Figure 3:**
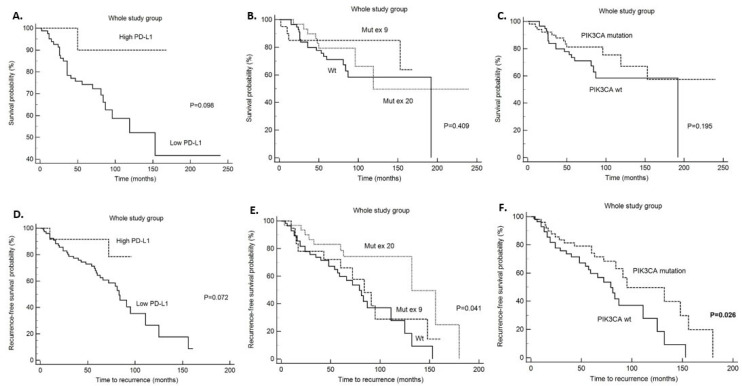
Disease specific survival (DSS), (**A**)—DSS depending on PD-L1 expression, (**B**)—DSS depending on presence of exon 9 and exon 20 *PIK3CA* mutation, (**C**)—DSS depending on presence of *PIK3CA* mutation; Disease-free survival (DFS), (**D**)—DFS depending on PD-L1 expression, (**E**)—DFS depending on presence of exon 9 and exon 20 *PIK3CA* mutation, (**F**)—DFS depending on presence of *PIK3CA* mutation (whole study group).

Disease specific survival (DSS) analysis has shown that patients without and with *PIK3CA* mutation with high CD4 tumor infiltrate had significantly better survival in comparison to patients with low CD4 infiltration (*p* = 0.015 and *p* = 0.0017, respectively) (Table 6). Also, we found that patients without *PIK3CA* mutation and high CD8 infiltrate had significantly better survival (*p* = 0.024), but the same was not found in patients with *PIK3CA* mutation (0.864) (Table 6 and Figure 1). All other survival analysis for DSS did not reach statistical significance in the *PIK3CA* non-mutated and mutated tumors (Table 6). When looking at the whole study group, patients with high CD4 and CD8 tumor infiltrates had a better prognosis with significantly longer DSS (*p* = 0.0003 and *p* = 0.041, respectively) (Table 6) and statistical trend to a better survival in the PD-L1 positive patients (*p* = 0.098) (Table 6). When looking at disease-free survival (DFS) in the non-mutated *PIK3CA* group and tumors with high CD4 infiltration, there was a statistically significant five-year survival without recurrences of 78.5% (*p* = 0.01) (Table 7). Also, in the *PIK3CA* mutated group and tumors with high CD4 infiltration, the five-year DFS rate was longer, (85% for CD4 high and 72% for CD4 low), and difference was statistically significant (*p* = 0.0458) (Table 7). Also, a statistical trend toward longer DFS was noticed in the *PIK3CA* mutated group and CD163 high infiltration (*p* = 0.077 and Figure 2). In the whole study group, when looking at DFS, statistical significance was shown in the patients with tumors with high CD4 (*p* = 0.005) and CD163 (*p* = 0–009) infiltrate (Table 7). In the patients with *PIK3CA* mutation and specifically exon 20 *PIK3CA* mutation, there was statistically significant longer survival without recurrence (*p* = 0.026 and *p* = 0.041, respectively) and statistical trend to a better DFS in PD-L1 positive patients (*p* = 0.072) (Table 7 and Figure 3). Also, we performed multivariate analysis for all the variables that reached statistical significance in the univariate analysis, and only *PIK3CA* in DFS retained statistical significance (*p* = 0.0488) (Table 8).

## 3. Discussion

In the early breast carcinoma, *PIK3CA* mutations have been detected in 37% of HR+/HER2− and associated with improved DFS but contrary *PIK3CA* mutations have been detected in 28% of HR+/HER2− metastatic breast carcinoma patients and correlated with worse overall survival as well as resistance to chemo- and endocrine therapy [18,24]. Results from our research are in agreement with previous studies because we also found 43.1% *PIK3CA* mutated tumors in HR+/HER2− breast carcinomas with majority having mutations in exon 20 (26.8%) and exon 9 (16.3%). Also, in our study group we found that *PIK3CA* mutations are associated with better DFS, but we did not find correlation between mutational status and DSS which corresponds to earlier findings. In addition, in this research we found that tumors with exon 20 *PIK3CA* mutations were more frequently associated with histologically more favorable characteristics like lower grade tumors, tumors with Ki67 < 20%, and patients had less frequently metastasis/recurrence during follow up. In this study, tumors with *PIK3CA* mutation had higher expression of PD-L1 in comparison to tumors without *PIK3CA* mutation but the difference did not reach statistical significance. However, the statistical significance was found in the distribution of PD-L1 positive tumors, when we compared the exon 9 and exon 20 *PIK3CA* mutated to the wild type (non-mutated) group, where patients with exon 20 mutations had higher number of PD-L1 positive tumors. This correlates with the literature data where *PIK3CA* mutations enhance transcription of PD-L1 and through AKT-mediated phosphorylation of β-catenin activate PD-L1 expression [25]. Also finding is opposite to research of Mosele et al. because they could not find any association between PD-L1 expression and *PIK3CA* mutations, but this is in TNBC, and they suggested that there is a population in which PI3K inhibitors could be developed independently from anti-PD-L1 agents [18]. However, data from the literature indicate that *PIK3CA* mutation might serve as a biomarker of an improved response to immune therapy in different types of carcinomas, like bladder, gastric, and cervical cancer [21,26,27]. Jiang W et al. showed a case where continuous pembrolizumab monotherapy treatment induced complete remission of a recurrent cervical cancer patient with systemic metastasis and *PIK3CA*-E545K mutation, implying that *PIK3CA* mutation is potentially a biomarker for pembrolizumab treatment in cervical cancer [21]. Having this in mind, data from our study might indicate that HR+/HER2− breast cancer patients with *PIK3CA* exon 20 mutation and PD-L1 expression might have the most benefit from immune checkpoint blockade. Also, all of the above findings from this study suggest that mutations in *PIK3CA* gene particularly in exon 20 are prognostically good characteristic in HR+/HER-2- breast carcinoma. Ruffell et al. observed that breast cancer tissues contained infiltrates dominated by CD8+ and CD4+ lymphocytes, with minor populations of NK cells and B lymphocytes, whereas in the normal breast tissue, myeloid-lineage cells including macrophages, mast cells, and neutrophils were more evident [28,29]. Despite the presence of many investigations, it is not possible to reach a clear and uniform conclusion about the role of each T-cell subset as well as M2-polarized (CD163+) macrophages in the breast TME or its association with breast carcinoma outcome [30]. Sobral-Leite et al. suggest that PI3K pathway alterations might be associated with the composition of the TME in luminal breast cancer, including the attraction of CD8-positive T-cells [20]. In their study, ER-positive breast cancer, with high tumor CD8 infiltration was associated with *PIK3CA* mutations and worse recurrence free survival and these associations were more pronounced among patients with grade 1 or 2 tumors [20]. In our study, we did not find statistical significance regarding DFS and CD8 lymphocyte infiltration in the non-mutated and mutated *PIK3CA* group but DSS was statistically significantly longer in CD8 high infiltration group in non-mutated *PIK3CA* patients indicating that wild type and mutated *PIK3CA* HR+/HER2− tumors have different functional state of the immune cells within TME indirectly supporting the findings of Sobral-Leite et al. Additionally, we did not find difference in the distribution of CD4, CD8, CD68, and CD163 infiltration between wild type (non-mutated) and *PIK3CA* mutated HR+/HER2− tumors. We thought that reason for this might be the fact that HR+/HER2− tumors are biologically heterogeneous group comprised from luminal A and B breast carcinoma. Thus, we additionally compared the immune cells composition between non-mutated and mutated *PIK3CA* luminal A and B breast carcinoma, but we did not find a statistically significant difference. However, when we compared the distribution of immune cells in wild type and *PIK3CA* mutated tumors who had metastasis/recurrence we found a statistically significant distribution of CD4 infiltration in wild type tumors (non-mutated) and CD163 infiltration in *PIK3CA* mutated tumors. Also, in the group of tumors with *PIK3CA* mutation and without metastasis/recurrence, there was a higher number of PD-L1 positive tumors but the result was not statistically significant (Table 4). The interesting finding is that when we analyzed CD4 infiltration and DSS or DFS, CD4 high infiltration in non-mutated and mutated *PIK3CA* group had better five -year survival, while the same was not found for CD8 infiltration in non-mutated and mutated *PIK3CA* tumors. These findings suggest that CD4 and CD8 TILs might have different functions and responses in *PIK3CA* mutated and non-mutated HR+/HER2− tumors status, so the final result of their complex interplay has a different impact on recurrence and overall survival. Within TME, tumor derived signals recruit monocytes and induce their polarization into tumor associated macrophages (TAMs) (M2 macrophages), promoting tumor cell proliferation, epithelial–mesenchymal transition (EMT), and suppression of CD8+ T-cell mediated anti-tumor effects [31]. So M2 macrophages facilitate tumor progression [31]. In this study, we found that *PIK3CA* mutated and non-mutated HR+/HER2− breast carcinomas had different correlations between PD-L1 expression and immune cells within TME (Table 5). An interesting finding is that CD163 as a marker of infiltration with M2 polarized macrophages (pro-tumor effect) in non-mutated *PIK3CA* group had a negative correlation with PD-L1 expression, while in the *PIK3CA* mutated group, this correlation was positive (Table 5). And also, when looking at the DFS in our study, patients with the *PIK3CA* mutation and high CD163 infiltration did not have disease recurrence (result was at the level of statistical trend, Table 7). The immunosuppressive properties of TAMs are generally believed to be dependent on the phosphatidylinositol 3-kinase (PI3K) signaling. High levels of M2 macrophages, particularly in the TME, are generally associated with poorer DFS but, nevertheless, a prognostic association in the opposite direction has also been suggested in breast cancer patients, thus further highlighting the need for a more in-depth evaluation of the TAM prognostic value [32]. Our result that shows high CD163 infiltrates to be associated with better DFS is contradictory, but at the same time it can be explained by new data in the literature where TAMs with dual characteristics of M1 and M2 have been identified, indicating the binary classification may be oversimplified. For example, Caronni et al. performed single-cell RNA-sequencing (scRNA-seq) on pancreatic ductal adenocarcinomas biopsies from cancer patients and revealed that inflammatory IL-1β + TAMs were shown to co-express both inflammatory (MHCII, CD80, and CD86) and immune inhibitory markers (CD206, arg-1, and PD-L1) [33]. This further emphasizes the complex interactions and crosstalk between immune cells and need for more precise TAM classification methods to help with the understanding of their dynamic functions. Also, this might further support the notion that *PIK3CA* mutations in HR+/HER2− breast tumors might influence and induce different functions of immune cells with prognostic result opposite from one expected knowing the pro-tumorigenic effects of M2 macrophages. Finding the explanation and mechanism on how mutations in *PIK3CA* achieve opposite effect of favorable versus poor prognosis and their effect on TME will be important in understanding the pathogenesis and progression of breast carcinoma. Also, this might have an impact on how to determine therapeutic approaches for patients with *PIK3CA* mutations in different stages and subtypes of disease. This emphasizes the need for more research regarding immune cells and *PIK3CA* mutations, especially in the era when there is available targeted therapy.

Limitations of this research would be the retrospective aspect of the study leading to heterogenic systemic treatment, but since all patients were treated at same clinical hospital within certified breast center, treatment decisions were made according to the national guidelines. Other limitations are the use of TMAs and *PIK3CA* mutation analysis using allele specific PCR detecting only “hot-spot” mutations. Using this type of assay, *PIK3CA* gene mutations, not covered by the kit, would not be detected, even though it remains difficult to interpret the functional consequences of new genetic mutations. Also, in order to compensate usage of TMA immunohistochemically stained slides, we used three to four 1 mm tissue cores as well as serial sections of the same TMA.

In conclusion, the focus of this research was HR+/HER2− breast carcinoma because studies examining *PIK3CA*, PD-L1 expression and immune cells within TME in this subtype are scarce. This study showed statistically significant distribution of PD-L1 expression in HR+/HER2−tumors with exon 20 *PIK3CA* mutation with different correlations of PD-L1 expression and immune cells infiltration within TME in mutated and non-mutated *PIK3CA* tumors. Currently, breast cancer with *PIK3CA* mutation comes into focus because novel research data in different cancer types indicate that mutations within *PIK3CA* might serve as a biomarker of an improved response to immune therapy.

## 4. Materials and Methods

### 4.1. Patients and Tumor Specimens

This retrospective study included 123 breast cancer samples over the period from 2010 to 2015 obtained from the archives of the Clinical Department of Pathology and Cytology, Clinical Hospital Center Rijeka, Croatia. The biopsy samples included hormone receptor (HR) positive/HER-2 negative breast cancer tissue from female patients that were not previously treated with radio or chemotherapy. The American Society of Clinical Oncology (ASCO)/College of American Pathologists (CAP) guideline recommendations were used as references for categorizing Ki-67, ER, progesterone receptor (PR), and HER2 status as part of the routine pathologic evaluation [34,35]. Also, the majority of material was surgical biopsy samples, but few were core biopsies that had enough tumor material. Clinicopathological parameters including age at initial diagnosis, tumor size, histologic grade, histologic subtype, lymphovascular and perineural invasion, axillary lymph node status, and clinical stage were obtained from the patient’s medical records. All carcinomas were classified according to the criteria of the WHO [36]. All of the patient’s clinicopathological characteristics are shown in more detail in Table 1. The study was conducted in accordance with the Declaration of Helsinki. The study was approved by the Institutional Ethical Committees of the Clinical Hospital Center Rijeka, Croatia class No:003-05/21-1/105, Ur.No:2170-29-02/1-21-2 and Faculty of Medicine, University of Rijeka, Croatia class No:003-08/21-01/75, Ur.No:2170-24-04-3-21-8 on 28 December 2021. Individual consent for this retrospective analysis was waived in accordance with the national legislation and the institutional requirements.

### 4.2. Immunohistochemical Staining

The tissue microarrays (TMAs) were constructed using three or four 1 mm cores of the above-mentioned archived biopsy samples. Also, to compensate for the spatial distribution of examined markers, we used serial sections of the same TMA cores. During immunohistochemical procedures, some cores were either lost, fragmented, or showed suboptimal staining; therefore, the number of examined samples sometimes differed between analyses. The antibodies used in this research were as follows: rabbit monoclonal antibody (IgG) anti-PD-L1 (clone SP142, Ventana, Tucson, AZ, USA), mouse monoclonal antibody (IgG) anti-CD4 (clone SP35, Cell Marque, Rocklin, CA, USA), mouse monoclonal antibody (IgG1) anti-CD8 (clone C8/144B, DakoAgilent, Santa Clara, CA, USA), mouse monoclonal antibody (IgG) anti-CD68 (clone PG-M1, DakoAgilent, Santa Clara, CA, USA), mouse monoclonal antibody (IgG1) anti-CD163 (clone 10D6, Leica Biosystems, Buffalo Grove, IL, USA). The antigen retrieval protocol, incubation, and other procedural steps included in the immunohistochemical analysis were conducted according to the guidelines provided by the manufacturer.

### 4.3. Immunohistochemical Evaluation

The independent evaluation of the expression of investigated biomarkers was conducted by two pathologists. Percentage of *PD-L1* expression in tumor infiltrating immune cells (IC) was assessed as the proportion of tumor area occupied by *PD-L1* positive immune cells of any intensity and in any cell compartment (membrane or cytoplasmic) [37]. TMA cores that contained less than 100 viable tumor cells were excluded from evaluation. For each of these percentages 1% or greater (≥1%) was considered positive score and less than 1% (<1%) as negative score. Results were evaluated with known positive and negative tissue controls.

The expression of CD4, CD8, CD68, CD163 was evaluated in three separate „hot spots” containing the highest density of immune cells and the number of immunoreactive cells per 0.4 mm × 0.4 mm was counted at ×200 magnification (×20 objective) [38]. The final number was calculated based on the average number of cells in three areas and expressed as the number of positive cells. For the statistical analyses, the number of CD4, CD8, CD68, and CD163 positive cells was divided into lower and higher groups based on ROC calculated cut-off values.

### 4.4. DNA Isolation from FFPE

Total DNA was isolated from formalin fixed paraffin embedded (FFPE) tumor tissue. Sample sectioning was conducted under great care, using procedures to avoid the risk of cross contamination between the samples. Depending on the amount of biopsy material embedded in paraffin, 4–10 sections (5 µm thick) were placed on microscopic slides for macrodisection. On a corresponding HE slide, the area with sufficient amount of tumor tissue was marked with a pen. The selected area of interest was then transferred and circled on unstained sections for macrodissection. The pieces of selected tumor tissue were scraped from the microscopic slides and put into microcentrifuge tube. The scrapped fragments were deparaffinized by adding 1 mL of xylene and heating at 55 °C for 30 min, followed by centrifugation and subsequent removal of the supernatant. Upon dewaxing with three washes of xylene, 1 mL of 100% ethanol was added to remove residual xylene. The tissues were dried at 37 °C for 30 min and DNA was isolated using NucleoSpin Tissue kit (Macharey-Nagel, Duren, Germany) according to the manufacturer’s instructions. Yield and the quality of isolated DNA was determined using Qubit 3.0 (ThermoFisher, Waltham, MA, USA).

### 4.5. PIK3CA Mutation Analysis

The isolated tumor DNA was analyzed for *PIK3CA* mutations using the Cobas *PIK3CA* mutation test (Roche Molecular Systems, Inc., Branchburg, NJ, USA) and Cobas z480 analyzer (Roche Diagnostics, Indianapolis, IN, USA) according to the manufacturer’s instructions. This real-time PCR method can detect mutations in 5 exons of the *PIK3CA* gene. The following mutations were detected using this mutation test in *PIK3CA* gene: exon 1: R88Q; exon 4: N345K; exon 7: C420R; exon 9: E542K, E545X (E545A, E545D, E545G, E545K), Q546X (Q546E, Q546K, Q546L, Q546R) and exon 20: M1043I, H1047X (H1047L, H1047R, H1047Y), G1049R. In 11 samples, due to poor quality of DNA, the status of *PIK3CA* gene could not be determined so the result was marked as invalid.

### 4.6. Statistical Analysis

The statistical analysis was conducted using MedCalc for Windows, version 23.3.4 (Med-Calc Statistical Software bvba in Ostend, Belgium). Frequency differences between nominal variables were assessed using Fisher’s exact test and chi-square test. Spearman’s rank correlation analysis was used to determine the association between PD-L1 and immune cells. The analysis of tumor recurrence prediction was performed using logistic regression. The Kaplan–Meier method was used to compute cumulative survival probability. The disparities in survival rates were assessed using a log-rank test. Multivariate analysis was performed using the Cox multiple-hazards model and *p*-value of 0.05 in the univariate survival analysis was adopted as the limit for inclusion in the multivariate model. All tests conducted were two-tailed, and a statistically significant result was defined when *p* < 0.05.

A receiver operating characteristic (ROC) curve was generated to evaluate the efficacy of CD4, CD8, CD68, and CD163 as biomarkers for predicting patient outcomes and determining the most effective statistical cut-off values. Hence, the ROC curve and Youden index were computed to optimize the sensitivity and specificity of the individual marker in predicting overall DSS and DFS in the univariate model. The area under the ROC curve (AUC) was calculated to assess the prediction model’s quality, along with a 95% confidence interval (CI). ROC analysis showed statistically significant cut-off values for CD4 (low CD4 versus high CD4 cut off value ≤6) (*p* = 0.001, AUC 0.711) and CD8 (low CD8 versus high CD8 cut off value < 8) (*p* = 0.014, AUC = 0.646). ROC calculated cut off values for CD68 (cut off value ≤ 23) and CD163 (cut off value > 12) did not show statistical significance (Supplementary Data Table S2). DSS was expressed as the number of months from diagnosis to the occurrence of a breast cancer related death. DFS was defined as the time interval from the date of diagnosis to the date of documented first recurrence of disease. If there was no recurrence, disease-free survival was determined as the date of last follow-up.

## Figures and Tables

**Table 1:** Patient and tumor characteristics.

Clinicopathological Parameters	Number of Patients (%)
Age (years) ≤50 >50	27 (22.0) 96 (78.0)
Histological grade	
1	50 (40.7)
2	61 (49.4)
3	4 (3.3)
N/A	8 (6.4)
Tumor size (cm) <2 ≥2 N/A	39 (31.7) 79 (64.2) 5 (4.1)
Multifocality Absent Present N/A	105 (85.4) 14 (11.4) 4 (3.2)
Exulcerated tumor Absent Present N/A	112 (91.1) 7 (5.7) 4 (3.2)
Breast Right Left Both	63 (51.6) 54 (44.3) 5 (4.1)
Inflammatory carcinoma Absent Present N/A	116 (94.3) 2 (1.6) 5 (4.1)
Histological type NST Spec.type (lobular) Other spec. type (papillary, mucinous, cribriform) Mixed NST + mucinous Mixed NST + lobular	90 (73.2) 20 (16.3) 8 (6.5) 4 (3.3) 1 (0.7)
Histological subtype Luminal A Luminal B Multifocality	65 (52.8) 57 (46.3) 1 (0.9)
Clinical stage 1 2 3 4 N/A	32 (26.0) 39 (31.7) 28 (22.8 21 (17.1) 3 (2.4)
Lymphovascular invasion Absent Present N/A	36 (29.3) 82 (66.7) 5 (4.0)
Perineural invasion Absent Present N/A	59 (48.8) 55 (45.5) 7 (5.7)
Necrosis Absent Present N/A	88 (71.5) 33 (26.9) 2 (1.6)
Calcifications Absent Present N/A	82 (66.7) 39 (31.7) 2 (1.6)
Ki-67 <20% ≥20%	72 (58.5) 51 (41.5)
Lymph node status Negative Positive N/A	51 (41.5) 54 (43.9) 18 (14.6)
Extra-nodal tumor spreading Absent Present N/A	21 (38.9) 32 (59.3) 1 (1.8)
Metastasis present at the diagnosis Absent Present N/A	99 (80.5) 22 (17.9) 2 (1.6)
Metastasis and recurrence Absent Present, local Present, distant metastasis N/A	63 (51.2) 8 (6.5) 46 (37.4) 6 (4.9)
Months until the appearance of recurrence or metastasis Median (range)	41.5 (3–180)
Follow up (months) Median (range)	73 (12–240)
Number of died patients N (%)	38 (30.9)
*PIK3CA* mutations Wild type Mutations Invalid	59 (48.0) 53 (43.1) 11 (8.9)
*PIK3CA* mutations Wild type Exon 9 Exon 20 N/A	59 (48.0) 20 (16.3) 33 (26.8) 11 (8.9)
PD-L1 <1% ≥1% N/A	85 (69.1) 12 (9.8) 26 (21.1)
CD4 median (range) CD8 median (range) CD68 median (range) CD163 median (range)	8.0 (0.0–232.0) 10 (0.0–142.0) 7 (1.0–75.0) 6 (0.0–68.0)

NST-no special type; N/A-not available.

**Table 2:** Comparison of HR+/HER2− tumor characteristics depending on *PIK3CA* mutational status.

Characteristics (HR+/HER-2-)	*PIK3CA*		*p*-Value	*PIK3CA*			*p*-Value
	Wt N (%)	Mt N (%)		Wt N (%)	Ex9 N (%)	Ex20 N (%)	
**Age (years)** ≤50 >50	14 (23.7) 45 (76.3)	12 (22.6) 41 (77.4)	1.00 ^¶^	14 (23.7) 45 (76.3)	6 (30.0) 14 (70.0)	6 (18.2) 27 (81.8)	0.608 ^¶^
**Clinical stage** I II III IV	12 (20.3) 19 (32.2) 17 (28.8) 11 (18.6)	15 (30.0) 17 (34.0) 8 (16.0) 10 (20.0)	0.390 *	12 (20.3) 19 (32.2) 17 (28.8) 11 (18.6)	4 (21.1) 5 (26.3) 4 (21.1) 6 (31.6)	11 (35.5) 12 (38.7) 4 (12.9) 4 (12.9)	0.319 *
**Tumor size** <2 cm ≥2 cm	18 (30.5) 41 (69.5)	17 (35.4) 31 (64.6)	0.679 ^¶^	18 (30.5) 41 (69.5)	5 (29.4) 12 (70.6)	12 (38.7) 19 (61.3)	0.697 ^¶^
**Multifocality** Present Absent	48 (82.8) 10 (17.2)	47 (94.0) 3 (6.0)	0.084 ^¶^	10 (17.2) 48 (82.8)	1 (5.3) 18 (94.7)	2 (6.5) 29 (93.5)	0.199 ^¶^
**Exulcerated** Present Absent	54 (93.1) 4 (6.9)	47 (94.0) 3 (6.0)	1.00 ^¶^	4 (6.9) 54 (93.1)	1 (5.3) 18 (94.7)	2 (6.5) 29 (93.5)	0.969 ^¶^
**Inflammatory carcinoma** Present Absent	55 (96.5) 2 (3.5)	50 (100) 0 (0)	0.497 ^¶^	2 (3.5) 55 (96.5)	0 (0) 19 (100)	0 (0) 31 (100)	0.409 ^¶^
**Bilateral tumor** Present Absent	54 (91.5) 5 (8.5)	52 (100) 0 (0)	0.059 ^¶^	5 (8.5) 54 (91.5)	0 (0) 20 (100)	0 (0) 32 (100)	0.099 ^¶^
**Histological type** Ductal NST Lobular Other spec. type Mixed (duct.+lob.)	44 (74.6) 9 (15.3) 5 (8.5) 1 (1.7)	42 (79.2) 9 (17.0) 2 (3.8) 0 (0)	0.569 *	44 (74.6) 9 (15.3) 5 (8.5) 1 (1.7)	14 (70.0) 6 (30.0) (0) 0 (0)	28 (84.8) 3 (9.1) 2 (6.1) 0 (0)	0.370 *
**Histological subtype** Luminal A Luminal B	27 (45.8) 32 (54.2)	31 (58.5) 22 (41.5)	0.191 ^¶^	27 (45.8) 32 (54.2)	10 (50.0) 10 (50.0)	21 (63.6) 12 (36.4)	0.254 ^¶^
**Histological grade** 1 2 3	21 (36.8) 33 (57.9) 3 (5.3)	24 (51.1) 23 (48.9) 0 (0)	0.131 *	21 (36.8) 33 (57.9) 3 (5.3)	5 (29.4) 12 (70.6) 0 (0)	19 (63.3) 11 (36.7) 0 (0)	0.055 *
**Lymph node status** Negative Positive	22 (43.1) 29 (56.9)	23 (53.5) 20 (46.5)	0.408 ^¶^	22 (43.1) 29 (56.9)	7 (43.7) 9 (56.2)	16 (59.3) 11 (40.7)	0.373 ^¶^
**Extranodal tumor spreading** Absent Present	11 (37.9) 18 (62.1)	7 (36.8) 12 (63.2)	1.00 ^¶^	11 (37.9) 18 (62.1)	3 (33.3) 6 (66.7)	4 (40.0) 6 (60.0)	0.953 ^¶^
**Metastasis present at diagnosis** Absent Present	46 (79.3) 12 (20.7)	42 (80.8) 10 (19.2)	1.00 ^¶^	46 (79.3) 12 (20.7)	14 (70.0) 6 (30.0)	28 (87.5) 4 (12.5)	0.302 ^¶^
**Metastasis and recurrence** Absent Present	26 (46.4) 30 (53.6)	31 (59.6) 21 (40.4)	0.183 ^¶^	26 (46.4) 30 (53.6)	9 (45.0) 11 (55.0)	22 (68.7) 10 (31.2)	0.097 ^¶^
**Ki-67** <20% ≥20%	31 (52.5) 28 (47.5)	35 (66.0) 18 (34.0)	0.179 ^¶^	31 (52.5) 28 (47.5)	10 (50.0) 10 (50.0)	25 (75.8) 8 (24.2)	0.041 ^¶^

N—number; Wt—no *PIK3CA* mutation; Mt—with *PIK3CA* mutation; Ex9—exon 9; Ex20—exon 20, NST—no special type; ^¶^—Fischer’s exact test; *—χ^2^-test.

**Table 3:** Comparison of tumor microenvironment composition depending on *PIK3CA* mutational status.

Variable	*PIK3CA*			*p*-Value
	Wt	Mutation		
**CD4 median (range)** **CD4** Low (≤6) High (>6)	8.0 (0.0–94.0) 21 (41.2) 30 (58.8)	9.0 (0.0–232.0) 14 (34.1) 27 (65.9)		0.436 0.524
**CD8 median (range)** **CD8** Low (≤8) High (>8)	10.0 (0.0–100.0) 17 (34.7) 32 (65.3)	14.5 (1.0–142.0) 15 (35.7) 27 (64.3)		0.655 1.00
**CD68 median (range)** **CD68** Low (≤23) High (>23)	8.0 (1.0–30.0) 43 (84.3) 8 (15.7)	8.0 (1.0–75.0) 37 (92.5) 3 (7.5)		0.794 0.336
**CD163 median (range)** **CD163** Low (≤12) High (>12)	8.0 (1.0–68.0) 32 (72.7) 12 (27.3)	6.0 (0.0–41.0) 32 (82.1) 7 (17.9)		0.143 0.433
**PD-L1** <1% ≥1%	42 (93.3) 3 (6.7)	34 (81.0) 8 (19.0)		0.110
**PD-L1** <1% ≥1%	42 (93.3) 3 (6.7)	Ex9 14 (93.3) 1 (6.7)	Ex20 20 (74.1) 7 (25.9)	0.044

Wt—without *PIK3CA* mutation.

**Table 4:** Comparison of microenvironment cellular composition between HR+/HER2− tumors with and without metastasis/recurrence depending on *PIK3CA* mutational status.

Variable *PIK3CA* Wt	Metastasis/Recurrence		*p*-Value
	No	Yes	
**CD4**			
Low (≤6)	5 (23.8)	15 (55.6)	0.039
High (>6)	16 (76.2)	12 (44.4)	
**CD8**			
Low (≤8)	6 (27.3)	9 (37.5)	0.539
High (>8)	16 (72.7)	15 (62.5)	
**CD68**			
Low (≤23)	17 (81.0)	24 (88.9)	0.683
High (>23)	4 (19.0)	3 (11.1)	
**CD163**			
Low (≤12)	12 (63.2)	19 (86.4)	0.144
High (>12)	7 (36.8)	3 (13.6)	
**PD-L1**			
<1%	17 (89.5)	22 (95.7)	0.581
≥1%	2 (10.5)	1 (4.3)	
***PIK3CA* mt**			
**CD4**			
Low (≤6)	7 (26.9)	6 (42.9)	0.480
High (>6)	19 (73.1)	8 (57.1)	
**CD8**			
Low (≤8)	8 (28.6)	6 (46.2)	0.307
High (>8)	20 (71.4)	7 (53.8)	
**CD68**			
Low (≤23)	25 (96.2)	11 (84.6)	0.253
High (>23)	1 (3.8)	2 (15.4)	
		
**CD163**			
Low (≤12)	18 (72.0)	14 (100)	0.036
High (>12)	7 (28.0)	0 (0)	
**PDL-1**			
<1%	21 (75.0)	12 (92.3)	0.398
≥1%	7 (25.0)	1 (7.7)	
**Whole study group**			
**CD4**			
Low (≤6)	17 (31.5)	22 (50.0)	0.096
High (>6)	37 (68.5)	22 (50.0)	
**CD8**			
Low (≤8)	17 (29.8)	17 (42.5)	0.279
High (>8)	40 (70.2)	23 (57.5)	
**CD68**			
Low (≤23)	46 (90.2)	38 (88.4)	1.00
High (>23)	5 (9.8)	5 (11.6)	
**CD163**			
Low (≤12)	32 (64.0)	36 (92.3)	0.002
High (>12)	18 (36.0)	3 (7.7)	
**PD-L1**			
<1%	43 (81.1)	37 (94.9)	0.065
≥1%	10 (18.9)	2 (5.1)	

Wt—without *PIK3CA* mutation; Mt—with *PIK3CA* mutation.

**Table 5:** Correlation of PD-L1 expression in the whole group and in non-mutated (wt) and mutated (mt) *PIK3CA* HR+/HER2− tumors.

Variable		CD4	CD8	CD68	CD163
PD-L1 wt	r_s_	0.025	0.063	0.053	−0.317
	*P*	0.872	0.693	0.730	0.052
PD-L1 mt	r_s_	0.285	0.462	0.398	0.617
	*P*	0.078	0.0027	0.0134	<0.0001
PD-L1 whole group	r_s_	0.203	0.312	0.238	0.205
	*P*	0.051	0.0026	0.023	0.061

Wt—no *PIK3CA* mutation; Mt—with *PIK3CA* mutation; Correlations were evaluated using Spearman rank correlation coefficient.

**Table 6:** Disease specific survival in patients without and with *PIK3CA* mutation depending on immune cell infiltration and for the whole study group.

Variable	N	Died from Underlying Disease (N)	Five-Year Survival (%)	Average Value ± SD	95% CI	χ^2^	Log-Rank Test (*p* Value)
**Patients without *PIK3CA* mutation (wt)**							
**CD4**							
Low (≤6)	20	10	63	86.7 ± 16.2	54.9–118.6	5.92	0.015
High (>6)	30	6	85	127.5 ± 10.2	107.5–147.6		
**CD8**							
Low (<8)	16	8	56	89.3 ± 18.9	52.1–126.4	5.09	0.024
High (≥8)	32	7	85	124.3 ± 10.1	104.5–144.2		
**CD68**							
Low (≤23)	42	15	75	111.0 ± 11.2	89.1–132.9	1.256	0.263
High (>23)	8	1	85	132.3 ± 16.4	100.1–164.4		
**CD163**							
Low (≤12)	31	10	76	99.3 ± 13.3	73.3–125.4	0.722	0.395
High (>12)	12	3	75	120.5 ± 14.8	91.4–149.5		
**PD-L1**							
<1%	41	13	77	119.2 ± 10.7	98.1–140.2	0.553	0.457
≥1%	3	0	100	55.0 ± 0.0	55.0–55.0		
**Patients with *PIK3CA* mutation (mt)**							
**CD4**							
Low (≤6)	13	7	63	89.1 ± 17.9	54.4–123.8	9.88	0.0017
High (>6)	26	2	85	223.6 ± 11.2	201.7–245.4		
**CD8**							
Low (<8)	13	4	75	164.8 ± 30.1	105.9–223.7	0.029	0.864
High (≥8)	27	6	85	129.6 ± 13.4	103.4–155.8		
**CD68**							
Low (≤23)	36	9	79	158.3 ± 22.9	113.2–203.3	1.219	0.269
High (>23)	3	0	100	168.0 ± 0.0	168.0–168.0		
**CD163**							
Low (≤12)	32	8	84	164.2 ± 21.6	121.9–206.5	0.045	0.831
High (>12)	7	1	87	86.9 ± 5.7	75.7 – 98.0		
**PD-L1**							
<1%	32	9	80	148.8 ± 23.0	103.7–193.9	0.859	0.354
≥1%	8	1	89	153.3 ± 13.8	126.2–180.3		
**Whole study group**							
**CD4**							
Low (≤6)	39	20	62	85.8 ± 10.5	65.3–106.4	13.29	0.0003
High (>6)	61	11	85	196.6 ± 12.1	172.9- 220.2		
**CD8**							
Low (<8)	34	15	62	129.7 ± 20.7	89.2–170.2	4.19	0.041
High (≥8)	65	16	85	128.5 ± 8.3	112.2–144.7		
**CD68**							
Low (≤23)	86	28	72	148.6 ± 14.6	120.1–177.1	2.14	0.143
High (>23)	11	1	90	153.8 ± 13.5	127.4–180.2		
**CD163**							
Low (≤12)	69	22	77	144.5 ± 16.0	113.1–175.9	0.829	0.363
High (>12)	23	5	87	124.7 ± 9.9	105.2–144.2		
**PD-L1**							
<1%	82	27	75	144.4 ± 15.2	114.7–174.2	2.741	0.098
≥1%	12	1	90	156.2 ± 11.2	134.3–178.1		
***PIK3CA*** **specific mutation**							
Wt	58	19	72	132.6 ± 11.7	109.7–155.6	1.788	0.409
Exon 9	20	4	85	140.8 ± 12.8	115.8–165.9		
Exon 20	31	8	80	159.4 ± 29.9	110.6–208.2		
***PIK3CA*** **mutation**							
Wt	58	19	72	132.6 ± 11.7	109.7–155.6	1.697	0.195
Mutation	51	12	81	173.5 ± 16.7	140.7–206.3		

Wt—without *PIK3CA* mutation.

**Table 7:** Disease-free survival in patients without and with *PIK3CA* mutation and whole study group.

Variable	N	Disease Recurrence (N)	Five Year DFS (%)	Mean Value ± SD	95% CI	χ2	Log-Rank Test (*p*-Value)
**Patients without *PIK3CA* mutation (wt)**							
**CD4**						6.64	0.01
Low (≤6)	20	15	45	51.95 ± 8.84	34.63–69.3		
High (>6)	28	12	78.5	89.82 ± 7.25	70.35–109.3		
**CD8**						1.84	0.175
Low (<8)	15	9	50	51.68 ± 8.90	34.24–69.12		
High (≥8)	31	15	65	84.37 ± 8.78	67.16–101.59		
**CD68**						0.007	0.932
Low (≤23)	41	24	55	72.4 ± 7.81	57.09–87.7		
High (>23)	7	3	41	66.21 ± 12.34	42.02–90.4		
**CD163**						2.02	0.155
Low (≤12)	31	19	55	69.3 ± 9.1	51.5–87.1		
High (>12)	10	3	78	78.61 ± 8.56	61.8–95.4		
**PD-L1**						0.07	0.786
<1%	39	22	59	72.45 ± 7.38	57.99–86.91		
≥1%	3	1	65	40.0 ± 12.25	15.99–64.01		
**Patients with *PIK3CA* mutation (mt)**							
**CD4**						3.98	0.0458
Low (≤6)	13	6	72	64.1 ± 10.4	43.7–84.4		
High (>6)	26	8	85	112.3 ± 14.4	84.1–140.4		
**CD8**						0.313	0.576
Low (<8)	13	6	75	101.6 ± 18.92	64.52–138.7		
High (≥8)	27	7	88	83.41 ± 4.62	74.32–92.45		
**CD68**						1.714	0.190
Low (≤23)	36	11	82	109.2 ± 12.29	85.13–133.29		
High (>23)	3	2	66	64.67 ± 8.99	47.03–82.30		
**CD163**						3.136	0.077
Low (≤12)	32	14	80	96.1 ± 11.77	73.02–119.17		
High (>12)	7	0	100	93.0 ± 0.0	93.0–93.0		
**PD-L1**						1.943	0.163
<1%	32	12	80	97.75 ± 12.8	72.58–122.9		
≥1%	8	1	100	92.0 ± 3.65	84.84–99.16		
**Whole study group**							
**CD4**						8.09	0.005
Low (≤6)	39	22	43	61.9 ± 6.6	49.1–74.8		
High (>6)	58	22	78	98.4 ± 8.5	81.8–115.0		
**CD8**						1.493	0.222
Low (≤8)	33	17	60	85.1 ± 12.4	60.8–109.2		
High (>8)	63	23	73	90.8 ± 5.2	73.6–103.0		
**CD68**						0.367	0.545
Low (≤23)	84	38	68	86.9 ± 7.2	72.9–101.0		
High (>23)	10	5	50	66.3 ± 9.2	48.3–84.3		
**CD163**						6.874	0.009
Low (≤12)	68	36	60.5	80.3 ± 7.5	65.7–94.9		
High (>12)	21	3	89	87.7 ± 4.6	78.8–96.7		
**PD-L1**						3.229	0.072
<1%	79	37	73	83.9 ± 7.5	69.3–98.5		
≥1%	12	2	91	85.7 ± 7.2	71.6–99.8		
***PIK3CA *** **specific mutation**						6.375	0.041
Wt	56	30	65	77.9 ± 7.7	62.9–93.0		
Exon 9	20	11	72	85.8 ± 13.5	59.5–112.2		
Exon 20	31	10	82	125.0 ± 14.0	97.7 ± 152.4		
***PIK3CA *** **mutation**						4.971	0.026
Wt	56	30	62	77.9 ± 7.7	62.9–93.0		
Mutation	51	21	79.5	107.4 ± 10.9	85.9–128.8		

DFS—disease-free survival; wt—without *PIK3CA* mutation; mt—with *PIK3CA* mutation.

**Table 8:** Multivariate analysis of disease specific and disease-free survival.

Disease Specific Survival			
Variables (Cut Off)	HR	95% CI	*p*-Value
CD4 (≤6)	0.51	0.19–1.30	0.159
CD8 (<8)	0.61	0.23–0.1.59	0.313
PD-L1	0.20	0.02–1.62	0.133
**Disease-free survival**			
CD4 (≤6)	0.54	0.24–1.21	0.134
CD163 (>12)	0.35	0.09–1.24	0.351
PD-L1	0.83	0.18–3.76	0.835
*PIK3CA* mutation	0.45	0.21–0.99	**0.0488**

HR—hazards ratio; CI—confidence interval.

## Data Availability

The datasets analyzed during the current study are available from the corresponding author upon reasonable request.

## Supplementary Materials

The following supporting information can be downloaded at: https://www.mdpi.com/article/10.3390/ijms26199489/s1.

## Abbreviations

The following abbreviations are used in this manuscript:

DCIS	Ductal carcinoma in situ
DFS	Disease-free survival
DSS	Disease-specific survival
FFPE	Formalin fixed paraffin embedded
HR+/HER2−	Hormone receptor positive/human epidermal growth factor-2 negative
IHC	Immunohistochemistry
MAP	Mitogen activated protein
MDSCs	Myeloid-derived suppressor cells
NST	No special type
PD-L1	Programmed cell death ligand 1
ROC	Receiver operating characteristic
TAMs	Tumor associated macrophages
TILs	Tumor-infiltrating lymphocytes
TMAs	Tissue microarrays
TME	Tumor microenvironment
TNBC	Triple negative breast carcinomas
