# Supplementary material for: Hormone Receptor Positive/HER2 Negative Breast Carcinoma: Association of PIK3CA Mutational Status with PD-L1 and Tumor Cell Microenvironment and Their Prognostic Significance
Source: Int J Mol Sci. 2025 Sep 28;26(19):9489. doi: 10.3390/ijms26199489 (PMC12524508; doi:10.3390/ijms26199489)
Supplement: Supplementary file 1 [file ijms-26-09489-s001.zip › ijms-3856829-supplementary.pdf]

Supplementary Materials

Table S1. Comparison of tumor microenvironment composition depending on *PIK3CA* mutational status

Variable		*PIK3CA*		P-value
		**Luminal A wt**	**Luminal A mutation**	
**CD4**				
	Low (≤6)	8 (30.8)	9 (34.6)	1.00
	High (>6)	18 (69.2)	17 (65.4)	
**CD8**				
	Low (≤8)	9 (36.0)	8 (29.6)	0.667
	High (>8)	16 (64.0)	19 (70.4)	
**CD68**				
	Low (≤23)	21 (84.0)	23 (92.0)	0.667
	High (>23)	4 (16.0)	2 (8.0)	
**CD163**				
	Low (≤12)	17 (77.3)	19 (76.0)	1.00
	High (>12)	5 (22.7)	6 (24.0)	
**PD-L1**				
	<1%	23 (95.8)	21 (77.8)	0.103
	≥1%	1 (4.2)	6 (22.2)	
**Variable**		***PIK3CA***		**P value**
		**Luminal B wt**	**Luminal B mutation**	
**CD4**				
	Low (≤6)	13 (52.0)	5 (33.3)	0.332
	High (>6)	12 (48.0)	10 (66.7)	
**CD8**				
	Low (≤8)	8 (33.3)	7 (46.7)	0.505
	High (>8)	16 (16.7)	8 (53.3)	
**CD68**				
	Low (≤23)	22 (84.6)	14 (93.3)	0.636
	High (>23)	4 (15.4)	1 (6.7)	

**CD163**				
	Low (≤12)	15 (68.2)	13 (92.9)	0.115
	High (>12)	7 (31.8)	1 (7.1)	
**PD-L1**				
	<1%	19 (90.5)	13 (86.7)	1.00
	≥1%	2 (9.5)	2 (13.3)	

Wt-without *PIK3CA* mutation

Table S2. A receiver operating characteristic (ROC) calculated cut off values

Variable	Cut-off	P-value	AUC
CD4	≤ 6	<0.001	0.711
CD8	<8	0.014	0.646
CD68	≤ 23	0.463	0.547
CD163	>12	0.842	0.513

AUC-area under the curve
